# Supplementary material for: Ultra-high field fMRI identifies an action-observation network in the common marmoset
Source: Commun Biol. 2023 May 22;6:553. doi: 10.1038/s42003-023-04942-8 (PMC10202933; doi:10.1038/s42003-023-04942-8)
Supplement: Supplementary file 2 — Supplementary Information [file 42003_2023_4942_MOESM2_ESM.pdf]

Ultra-high field fMRI identifies an action-observation network in the common marmoset

Short Title:

Action-observation network in marmoset monkeys

Alessandro Zanini[1,*], Audrey Dureux[1], Janahan Selvanayagam[2], Stefan Everling[1,2]

Author affiliations:

[1]Centre for Functional and Metabolic Mapping, Robarts Research Institute, University of Western Ontario, London, Ontario, Canada

[2]Department of Physiology and Pharmacology, University of Western Ontario, London, Ontario, Canada

*Corresponding author:

Alessandro Zanini, Robarts Research Institute, University of Western Ontario, London, Ontario, Canada. **Email**: azanini4@uwo.ca

Supplementary Information

Supplementary Note 1 - Individual results and overlap across animals

To represent the variability of activations more thoroughly on an individual basis, Supplementary Figures 1-C and 2-C display the individual maps of the seven marmosets in our sample for the two main experimental contrasts of our study (Intact vs Scrambled Movement and Grasping vs Empty Hand, respectively). By creating an individual mask that includes only voxels with a z-score > 1.96 ($p < 0.05$ uncorrected) and summing the seven masks obtained using AFNI's 3dcalc function, we can observe the extent of overlap among individual results. This overlap is represented in panels A (left hemisphere) and B (right hemisphere) of Supplementary Figures 1 (Intact vs Scrambled Movement) and 2 (Grasping vs Empty Hand). In this representation, a voxel active for a specific contrast in all marmosets will have a value equal to 7, while a voxel never active will have a value of 0. Although the statistical power at the individual level is too low for quantitative analysis, this representation provides information that supports and extends previous observations.

For instance, in the contrast between Intact and Scrambled movement, Supplementary Figure 1 demonstrates that the left hemisphere activation lateralization is not consistent across all tested animals. While this pattern is reproduced in some animals (particularly M4 and M5), the extension and amplitude of activations in most marmosets appear comparable between the two hemispheres. Additionally, a bilateral prefrontal activation cluster similar in localization to that found in the comparison between goal-directed and non-goal-directed actions can be observed in M2, M4, M6, and M7. However, this cluster does not withstand the stricter statistical correction performed on the entire sample. Panels A and B of Supplementary Figure 1 confirm the presence of this prefrontal cluster, supporting the main text and Figure 1 observations: the greater overlap of individual activations is found at the prefrontal (particularly in areas 45 and 6Va) and occipito-temporal levels (with overlapping peaks in areas FST, PGa-IPa, and TE3), bilaterally.

Similarly, the individual maps of the goal-directed vs non-goal-directed actions comparison provide supporting information for previous descriptions. Supplementary Figure 2-C reveals that, in this case, the prefrontal cluster is more extensive and consistent across monkeys than in the previous contrast. This greater stability bolsters the involvement of this region in the marmosets' AON. In panels A and B, the overlapping regions of individual results are more extensive,

indicating greater consistency among these maps. Overlap peaks in the prefrontal region include the dorsal (6DR and 6DC) and ventral premotor areas (6Va), as well as prefrontal areas 45, 47, 8C, and 8Av. Posteriorly, activations of 5 out of 7 animals overlap in the left FST's most caudal part, but bilaterally, overlap of 3 or 4 marmosets can also be observed in the PGa-IPa, TE3, and V4T areas.

An interesting aspect, but that we cannot investigate in depth because of the composition of our sample, concerns the manual preference shown by our marmosets. As already amply demonstrated in literature[1–3], marmosets often show a strong manual preference in tasks of reaching for food, and such preference may reflect hemispheric dominance of other cognitive domains[4]. Unfortunately, only one (M3) of the 7 animals we tested shows a preference for the use of the left hand, and we are therefore unable to investigate statistically differences in the AON related to this preference. In addition, the lack of separation of videos representing actions carried out with the left hand and with the right hand in our experimental design adds a degree of uncertainty to any possible interpretation. On a purely qualitative level, the Supplementary Figures 1-C and 2-C do not seem to report a different pattern of activations for M3 compared to the 6 right-handed animals. The impossibility to test the relationship between the manual preference of marmosets and the extension/lateralization of their action observation network is therefore a limit of this study and a possible starting point for future work.

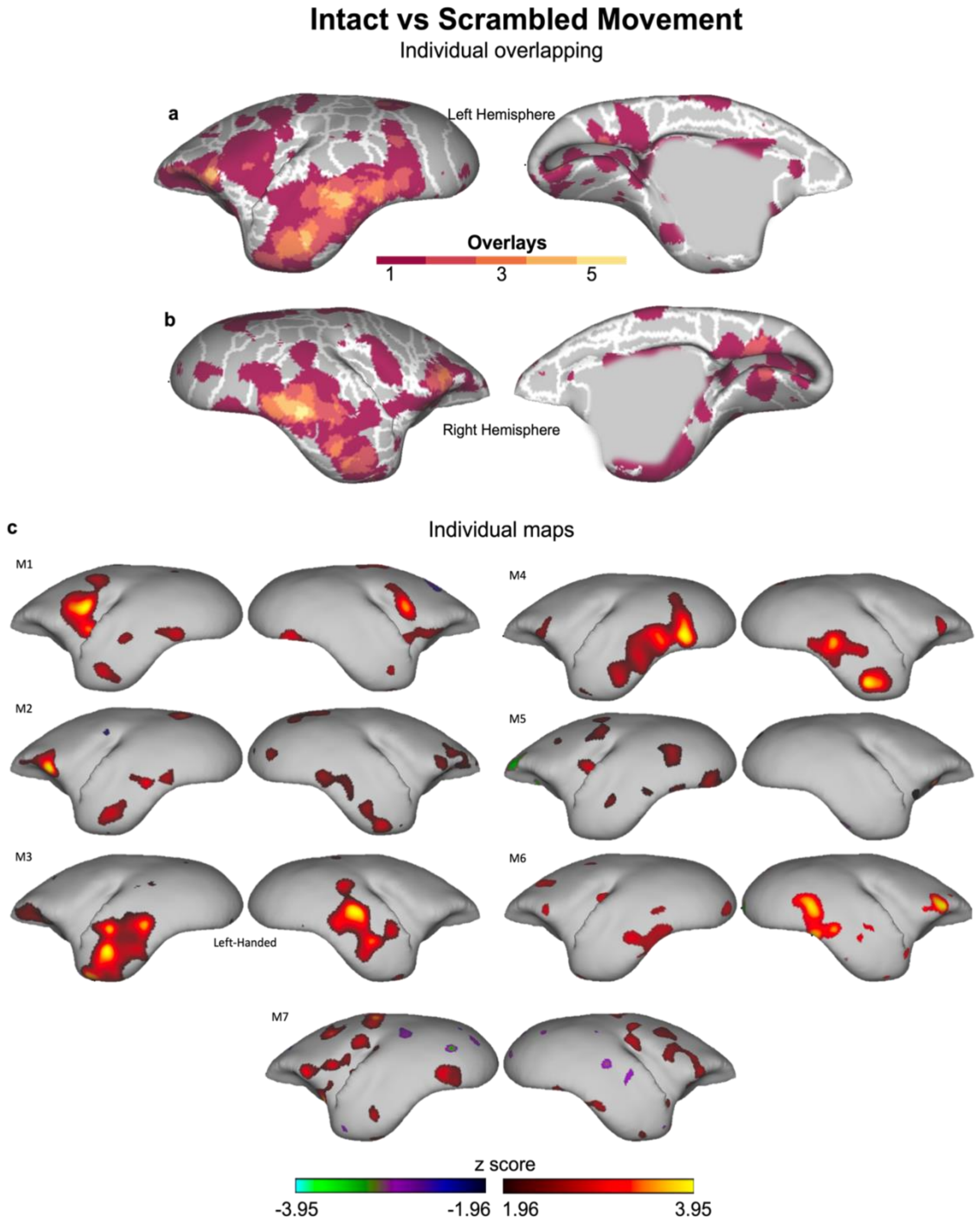

Supplementary Figure 1. Individual maps and their overlapping for the Intact vs Scrambled Movement comparison. In panel C, the results of the individual t-tests comparing Grasping Hand + Empty Hand videos versus Scrambled Grasping Hand + Scrambled Empty Hand ones. Results are reported at $p<0.05$ incorrected. Panels A (left hemisphere) and B (right hemisphere) show the overlap between the 7 individual maps: a value of 1 means that the voxel is activated only for one monkey, whereas a voxel activated by the same contrast in all monkeys will report a value of 7. White lines delineate the cerebral areas included in the Paxinos parcellation of the NIH marmoset brain atlas (Liu et al., 2018).

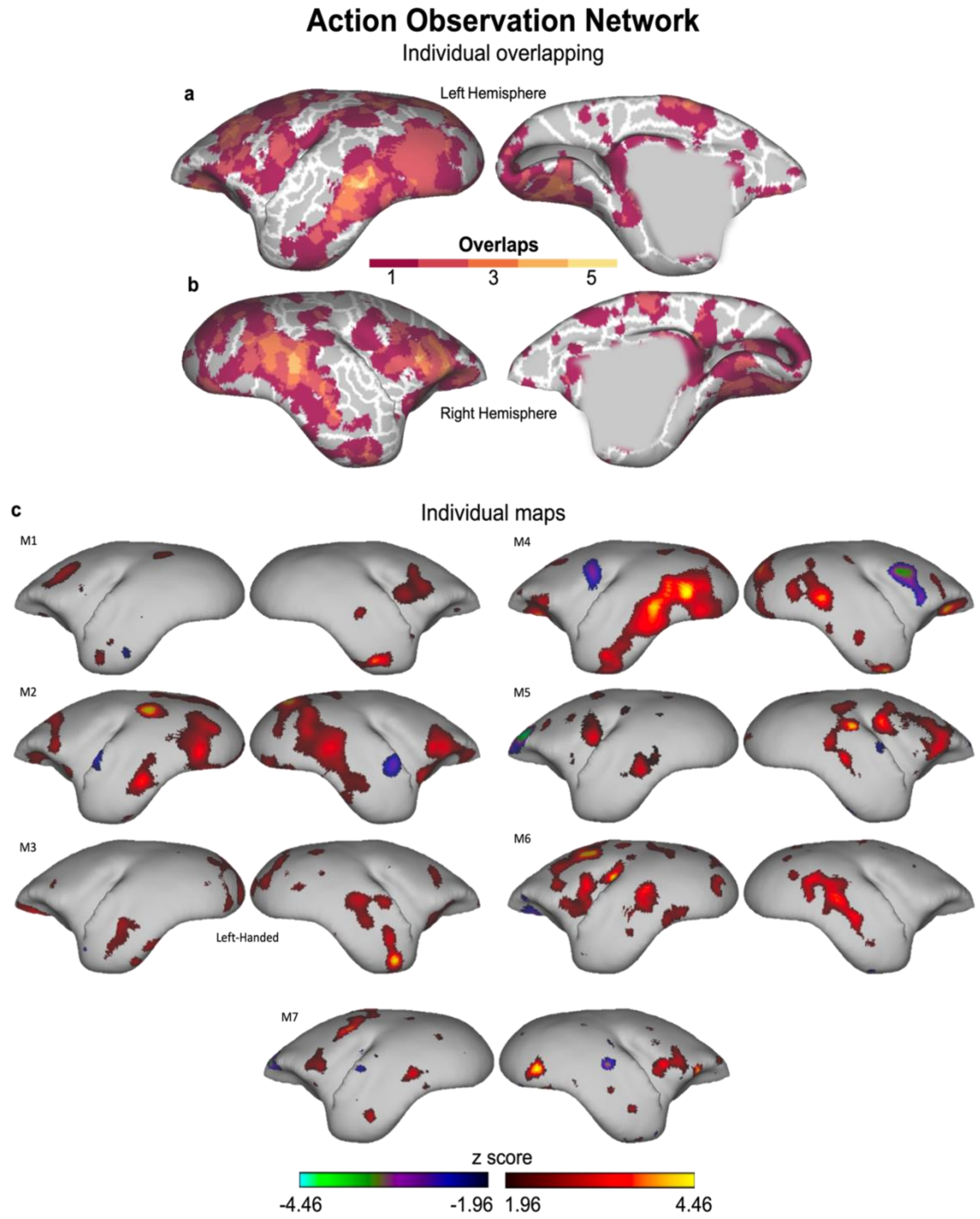

Supplementary Figure 2. Individual maps and their overlapping for the Grasping Hand vs Empty Hand comparison. In panel C, the results of the individual t-tests comparing Grasping Hand versus Empty Hand videos. Results are reported at $p<0.05$ incorrected. Panels A (left hemisphere) and B (right hemisphere) show the overlap between the 7 individual maps: a value of 1 means that the voxel is activated only for one monkey, whereas a voxel activated by the same contrast in all monkeys will report a value of 7. White lines delineate the cerebral areas included in the Paxinos parcellation of the NIH marmoset brain atlas (Liu et al., 2018).

Supplementary Note 2 - Action observation network and visual dorsal stream

Intrigued by the lack of activation in AIP within the action observation network of common marmosets, we conducted a qualitative comparison between the anatomical-functional connections of the dorsal visual stream areas (i.e., V6, V6A, LIP, MIP, and AIP) and the network of activations reported through the contrast between Grasping Hand and Empty Hand. Supplementary Figure 3 displays A) the action observation network of the common marmoset, B) the anatomical connectivity of the dorsal visual stream areas (Marmoset Brain Connectivity Atlas[5,6]) and C) the functional connectivity of these areas (Marmoset Connectome[7]). What emerges from this analysis may represent further evidence in favor of the inclusion of AIP in the action observation network of the common marmosets. Observing figure Supplementary Figure 3 (panel B), in fact, AIP is the only area of the dorsal visual stream to report a direct anatomical connection with the frontal areas included by us in the AON, and in particular with area 45 and the premotor region. While all the areas we analyzed exhibit functional connectivity that may parallel the fronto-temporo-parietal activations of the common marmoset AON, only injections into AIP revealed anatomical connections with the most frontal part of this network. This parallelism, combined with the absence of clear activation of AIP in our Grasping Hand vs Empty Hand contrast, raises important questions about the inclusion of this area within the AON. Further research is necessary to determine whether AIP could serve as the parietal node connecting the dorsal visual stream to the action observation network.

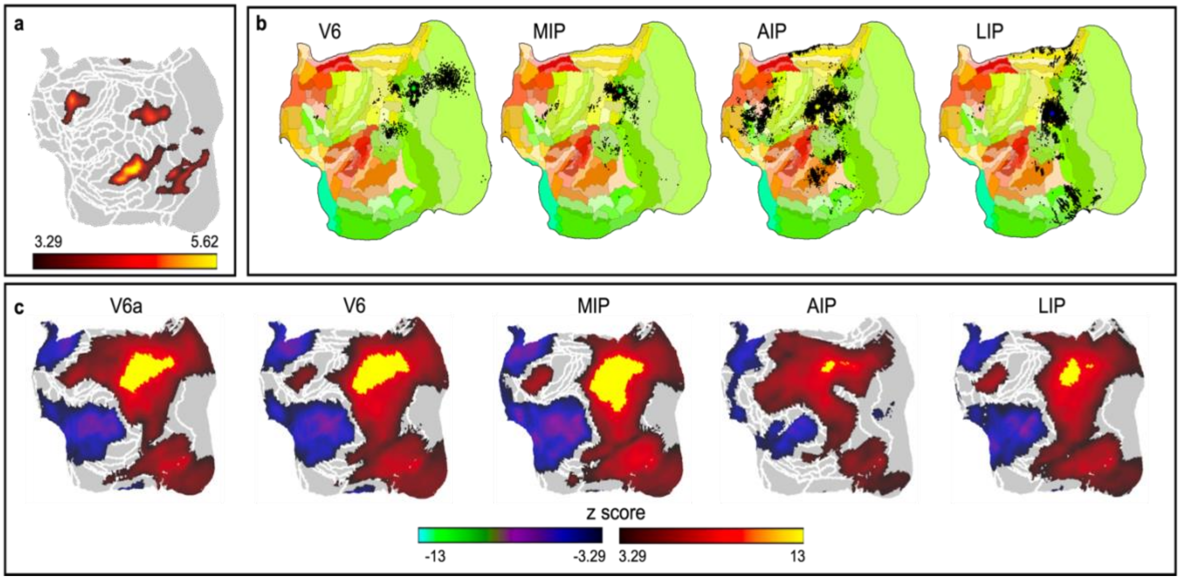

Supplementary Figure 3. Anatomical and functional connectivity of the areas of the dorsal visual stream in the common marmoset. In panel A, the action observation network, as obtained contrasting our Grasping Hand vs Empty Hand conditions. Results (z-score) are reported at p<0.001 and cluster-size corrected with a Monte-Carlo method. In panel B, the anatomical connections of the areas included in marmoset's dorsal visual stream, obtained through injections of anterograde tracers and available online (Marmoset Brain Connectivity Atlas[108, 109]). In panel C, the functional connectivity of these regions, obtained from fully awake marmosets and available online (Marmoset Connectome[110]).

Supplementary Note 3 - Single condition activation maps

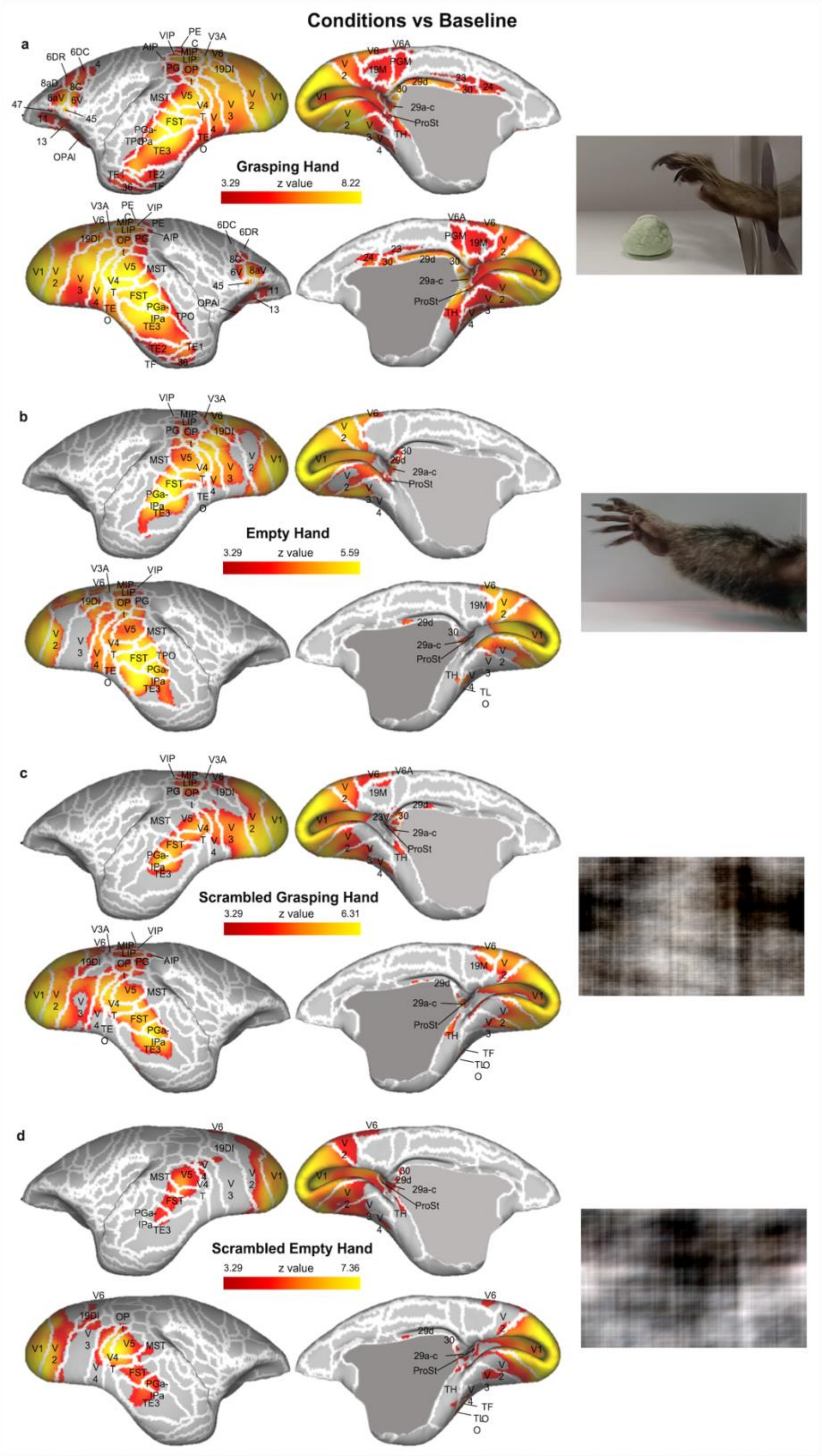

Supplementary Figure 4. Functional maps of the contrasts between each condition and baseline. Grasping Hand (A), Empty Hand (B), Scrambled Grasping Hand (C) and Scrambled Empty Hand (D) maps are displayed on the left and right fiducial brain surfaces (lateral view on the left, medial view on the right). White lines delineate the cerebral areas included in the Paxinos parcellation of the NIH marmoset brain atlas (Liu et al., 2018). The responses here reported have intensity higher than z = 3.29 (corresponding to p<0.001, AFNI's 3dttest++) and survived the cluster-size correction (10000 Monte-Carlo simulations, with a=0.05).

Supplementary Note 4 - Eye movement traces

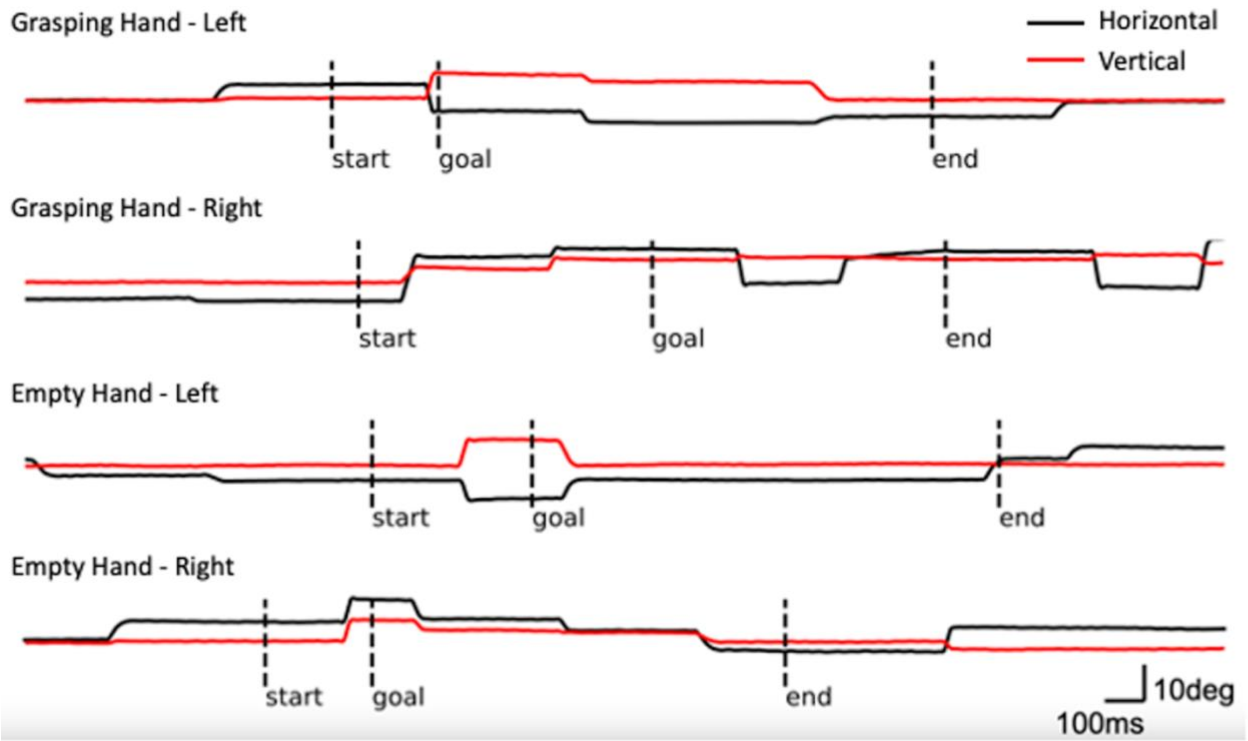

Supplementary Figure 5. Eye movement traces for Grasping and Empty Hand videos used in the eye-tracking experiment, for both actions performed from the left and from the right side of the screen. The black and red lines represent the horizontal and vertical components of eye movements respectively. For each type of video, the dashed lines represent three key moments: the beginning of the movement (the arm appears on the screen), the reaching of the goal (in Grasping Hand, grasping of the marshmallow, in Empty Hand, reaching the point of maximal extension of the arm for that specific video) and the end of the action (arm disappears from the screen).

Supplementary References

1. Cordeiro de Sousa, M. B., Xavier, N. S., Alves da Silva, H. P., Souza de Oliveira, M. & Yamamoto, M. E. Hand preference study in marmosets (Callithrix jacchus) using food reaching tests. *Primates* **42**, 57–66 (2001).
2. Rogers, L. J. Hand and paw preferences in relation to the lateralized brain. *Philos. Trans. R. Soc. Lond. B. Biol. Sci.* **364**, 943–954 (2009).
3. Hook, M. A. & Rogers, L. J. Development of hand preferences in marmosets (Callithrix jacchus) and effects of aging. *J. Comp. Psychol. Wash. DC 1983* **114**, 263–271 (2000).
4. Hook, M. A. & Rogers, L. J. Visuospatial reaching preferences of common marmosets (Callithrix jacchus): an assessment of individual biases across a variety of tasks. *J. Comp. Psychol. Wash. DC 1983* **122**, 41–51 (2008).
5. Majka, P. *et al.* Towards a comprehensive atlas of cortical connections in a primate brain: Mapping tracer injection studies of the common marmoset into a reference digital template. *J. Comp. Neurol.* **524**, 2161–2181 (2016).
6. Majka, P. *et al.* Open access resource for cellular-resolution analyses of corticocortical connectivity in the marmoset monkey. *Nat. Commun.* **11**, 1133 (2020).
7. Schaeffer, D. J. *et al.* An open access resource for functional brain connectivity from fully awake marmosets. *NeuroImage* **252**, 119030 (2022).
