# Supplementary material for: Ultra-high field fMRI identifies an action-observation network in the common marmoset
Source: Commun Biol. 2023 May 22;6:553. doi: 10.1038/s42003-023-04942-8 (PMC10202933; doi:10.1038/s42003-023-04942-8)
Supplement: Supplementary file 3 — Reporting Summary [file 42003_2023_4942_MOESM3_ESM.pdf]

Corresponding author(s): Alessandro Zanini

Last updated by author(s): Apr 28, 2023

Reporting Summary

Nature Portfolio wishes to improve the reproducibility of the work that we publish. This form provides structure for consistency and transparency in reporting. For further information on Nature Portfolio policies, see our Editorial Policies and the Editorial Policy Checklist.

Statistics

For all statistical analyses, confirm that the following items are present in the figure legend, table legend, main text, or Methods section.

n/a	Confirmed	
☐	☒	The exact sample size (n) for each experimental group/condition, given as a discrete number and unit of measurement
☐	☒	A statement on whether measurements were taken from distinct samples or whether the same sample was measured repeatedly
☐	☒	The statistical test(s) used AND whether they are one- or two-sided<br>*Only common tests should be described solely by name; describe more complex techniques in the Methods section.*
☐	☒	A description of all covariates tested
☐	☒	A description of any assumptions or corrections, such as tests of normality and adjustment for multiple comparisons
☐	☒	A full description of the statistical parameters including central tendency (e.g. means) or other basic estimates (e.g. regression coefficient) AND variation (e.g. standard deviation) or associated estimates of uncertainty (e.g. confidence intervals)
☐	☒	For null hypothesis testing, the test statistic (e.g. F, t, r) with confidence intervals, effect sizes, degrees of freedom and P value noted<br>*Give P values as exact values whenever suitable.*
☒	☐	For Bayesian analysis, information on the choice of priors and Markov chain Monte Carlo settings
☒	☐	For hierarchical and complex designs, identification of the appropriate level for tests and full reporting of outcomes
☒	☐	Estimates of effect sizes (e.g. Cohen's d, Pearson's r), indicating how they were calculated

Our web collection on statistics for biologists contains articles on many of the points above.

Software and code

Policy information about availability of computer code

Data collection	No software was used
Data analysis	Code supporting this study is available on OSF at https://osf.io/hvbmy/?view_only=6ea57106e8ce464fb0574a1acbc2f89d

For manuscripts utilizing custom algorithms or software that are central to the research but not yet described in published literature, software must be made available to editors and reviewers. We strongly encourage code deposition in a community repository (e.g. GitHub). See the Nature Portfolio guidelines for submitting code & software for further information.

Data

Policy information about availability of data

All manuscripts must include a data availability statement. This statement should provide the following information, where applicable:

- Accession codes, unique identifiers, or web links for publicly available datasets
- A description of any restrictions on data availability
- For clinical datasets or third party data, please ensure that the statement adheres to our policy

Data and code supporting this study are available on OSF at https://osf.io/hvbmy/?view_only=6ea57106e8ce464fb0574a1acbc2f89d

Human research participants

Policy information about studies involving human research participants and Sex and Gender in Research.

Reporting on sex and gender	No human participants involved in the study
Population characteristics	No human participants involved in the study
Recruitment	No human participants involved in the study
Ethics oversight	No human participants involved in the study

Note that full information on the approval of the study protocol must also be provided in the manuscript.

Field-specific reporting

Please select the one below that is the best fit for your research. If you are not sure, read the appropriate sections before making your selection.

☒ Life sciences ☐ Behavioural & social sciences ☐ Ecological, evolutionary & environmental sciences

For a reference copy of the document with all sections, see nature.com/documents/nr-reporting-summary-flat.pdf

Life sciences study design

All studies must disclose on these points even when the disclosure is negative.

Sample size	Sample size for fMRI study (n=7 common marmosets) and eye-tracking study (n=10, the 7 fMRI marmosets + 3 naives marmosets) was not calculated a priori, but determined by the availability of experimental animals in our laboratory. We suggest that this sample size is sufficient to verify our hypotheses as many other studies in the literature involving common marmosets and fMRI have used samples of the same or smaller size.
Data exclusions	No data excluded from the eye-tracking experiment. In the fMRI study, the compliance of the animal during each run was checked and noted online by the investigator; runs in which the animal closed its eyes for two or more stimulation blocks (regardless of the experimental condition) were discarded from analyses.
Replication	Quality of the signal was investigated across sessions and monkeys calculating the signal-to-noise ratio of each functional run. Too low signal-to-noise ratio may indicate a bad positioning of the coils, leading to poor/noisy results. The signal-to-noise ratio of the run selected for the fMRI experiment is stable and similar across sessions and monkeys.<br>A similar protocol, with similar experimental conditions, have been recently performed in our lab (data not published, manuscript in preparation). The results of this last experiment confirm and overlap with the action-observation network reported in the present manuscript.
Randomization	No randomization performed, due to the presence of only one experimental group.
Blinding	No blinding, due to no randomization of the experimental group.

Reporting for specific materials, systems and methods

We require information from authors about some types of materials, experimental systems and methods used in many studies. Here, indicate whether each material, system or method listed is relevant to your study. If you are not sure if a list item applies to your research, read the appropriate section before selecting a response.

Materials & experimental systems

n/a	Involved in the study	
☒	☐	Antibodies
☒	☐	Eukaryotic cell lines
☒	☐	Palaeontology and archaeology
☐	☒	Animals and other organisms
☒	☐	Clinical data
☒	☐	Dual use research of concern

Methods

n/a	Involved in the study	
☒	☐	ChIP-seq
☒	☐	Flow cytometry
☐	☒	MRI-based neuroimaging

Animals and other research organisms

Policy information about studies involving animals; ARRIVE guidelines recommended for reporting animal research, and Sex and Gender in Research

Laboratory animals	Seven common marmosets (Callithrix jacchus; three females, average age: 35.8±8.5 months, ranging from 30 to 54 months, average weight: 398 ± 46.7 g, ranging from 328 to 462 g) took part in the awake fMRI study. 10 common marmosets (5 females, average age 35.5±6.78 months, average weight 417.4±57.7 grams) took part in the eye-tracking experiment.
Wild animals	The study did not involve wild animals.
Reporting on sex	The samples of both experiments are composed of an equivalent number of male and female marmosets. The analyses reported in the manuscript were performed on the entire sample, without differentiation of sex. Findings in the action observation network literature do not suggest gender or sex differences.
Field-collected samples	The study did not involve samples collected from the field
Ethics oversight	All the experimental procedures described were performed in accordance with the guidelines of the Canadian Council on Animal Care policy on the care and use of experimental animals and an animal use protocol #2021-111 approved by the Animal Care Committee of the University of Western Ontario.

Note that full information on the approval of the study protocol must also be provided in the manuscript.

Magnetic resonance imaging

Experimental design

Design type	Task fMRI, with a block design
Design specifications	In each run, 9 baseline blocks (18 seconds long) were alternated with experimental blocks (12 seconds long) of four different conditions: Grasping Hand, Empty Hand, Grasping Hand Scrambled and Empty Hand Scrambled. Each run lasted 258 seconds globally. During baseline blocks, a black dot was presented at the center of the screen.
Behavioral performance measures	The task was a free-viewing task, presenting videos to the head-fixed animals. To confirm the compliance of the monkey, the percentage of time spent with eyes open in each run for each animals was computed.

Acquisition

Imaging type(s)	Functional
Field strength	9.4
Sequence & imaging parameters	For functional imaging, gradient-echo-based, single-shot echo-planar images covering the whole brain were acquired over multiple daily sessions (TR=1500 ms; TE=15 ms; flip angle=40°; FOV=64×48 mm; matrix size=96×128; voxel size=0.5 mm isotropic; number of slices=42 [axial]; bandwidth=400 kHz; GRAPPA acceleration factor (left-right=2). To correct for spatial distortion, a second set of echo-planar images with the opposite phase-encoding direction (right-left) was collected. To perform anatomical registration, a T2-weighted structural image was acquired for each animal with the following parameters: TR=7000 ms; TE=52 ms; FOV=51.20 × 51.20 mm; voxel size=0.133×0.133×0.5 mm; number of slices=45 (axial); bandwidth=50 kHz, GRAPPA acceleration factor=2.
Area of acquisition	Whole brain

Diffusion MRI ☐ Used ☒ Not used

Preprocessing

Preprocessing software	The data were preprocessed using a combination of AFNI and FSL's functions. The raw functional images were first converted to the NIfTI format using dcm2niix and then reoriented (FSL's fslswapdim and fslorient) to correct the sphinx posture. The reoriented functional images were preprocessed through procedures for eliminating any outliers (detected via AFNI's 3dToutcount), despiking (AFNI's 3dDespike) and time shifting (AFNI's 3dTshift). The images thus obtained were registered to the base volume (extracted at the half of each run and therefore corresponding to the 86th volume) using AFNI's 3dvolreg function. Then, all volumes were smoothed (AFNI's 3dmerge, FWHM Gaussian kernel of 2mm) and bandpass-filtered (AFNI's 3dBandpass, lowest frequency of 0.1 and highest frequency of 0.01). An average functional image was calculated for each run of each animal and then registered (FSL's FLIRT function) to the respective T2-weighted image. The transformation matrix thus obtained was subsequently used to carry out the 4D time series data. T2-weighted images were manually skull-stripped (removing the olfactory bulb) and the mask obtained was applied to the functional images.<br>Finally, anatomical and functional images were registered to the NIH marmoset brain atlas via the nonlinear registration operated by ANTs' (Advanced Normalization Tools) ApplyTransforms function.
Normalization	Anatomical and functional images of each animal were registered to the NIH marmoset brain atlas via the nonlinear registration operated by ANTs' (Advanced Normalization Tools) ApplyTransforms function.

Normalization template	NIH marmoset brain atlas
Noise and artifact removal	The reoriented functional images were preprocessed through procedures for eliminating any outliers (detected via AFNI's 3dToutcount), despiking (AFNI's 3dDespike) and time shifting (AFNI's 3dTshift).
Volume censoring	No volume censoring performed

Statistical modeling & inference

Model type and settings	The scan timing was convolved to the BOLD response (AFNI's 3dDeconvolve) specifying the 'BLOCK' convolution and extracting a regressor for each condition (Grasping Hand, Empty Hand and the two Scrambled conditions) for each run to be used in the subsequent regression analysis. All the conditions were entered in the model, along with polynomial detrending regressors (n=5). This regression generated four T-value maps, corresponding to the four experimental conditions, per animal per run, that were registered to the NIH marmoset brain atlas. These maps were thus compared through paired t tests (AFNI's 3dttest++).<br>At the group level, the T-maps of each animal for each condition and for each run (n=10) were converted into Z-maps. To investigate the neural responses induced by the perception of an "intact" biological movement versus a scrambled movement pattern, the Z score maps (n=140) of the Grasping and Empty Hand conditions of each animal were compared via t-test to those of the Scrambled Grasping Hand and Scrambled Empty Hand conditions. For protection against false positives, the results of this t-tests have been corrected with a minimum cluster-size resulting from 10000 Monte Carlo simulations (AFNI's 3dttest++ with Clustsim option, p<0.001 and alpha=0.05, nearest-neighbor clustering method 2, two-sided).<br>Finally, the presence of areas selective for goal-directed actions was investigated by comparing the Z value maps (n=70) of the Grasping Hand and Empty Hand conditions. As described above, we compared these maps using a paired t-test, which result was protected from false positives thanks to the same cluster-size correction (AFNI's 3dttest++ with Clustsim option, p<0.001 and alpha=0.05, nearest-neighbor clustering method 2, two-sided).
Effect(s) tested	Grasping Hand + Empty Hand versus Scrambled Grasping Hand + Scrambled Empty Hand (to investigate the neural responses induced by the perception of an "intact" biological movement versus a scrambled movement pattern)<br><br>Grasping Hand versus Empty Hand (to investigate the presence and extension of a putative action observation network in marmosets, responding more strongly to goal-directed actions compared non-goal-directed ones.

Specify type of analysis: ☒ Whole brain ☐ ROI-based ☐ Both

Statistic type for inference<br>(See Eklund et al. 2016)	Cluster-wise: AFNI's 3dttest++ with Clustsim option, p<0.001 and alpha=0.05, nearest-neighbor clustering method 2, two-sided
Correction	For protection against false positives, the results the t-tests have been corrected with a minimum cluster-size resulting from 10000 Monte Carlo simulations (AFNI's 3dttest++ with Clustsim option, p<0.001 and [illegible]=0.05, nearest-neighbor clustering method 2, two-sided).

Models & analysis

n/a	Involved in the study
☒	☐ Functional and/or effective connectivity
☒	☐ Graph analysis
☒	☐ Multivariate modeling or predictive analysis
